# Analysis of malingered psychological symptoms in a clinical sample for early detection in initial interviews

**DOI:** 10.1007/s00406-022-01422-8

**Published:** 2022-05-19

**Authors:** Carlos Barbosa-Torres, Natalia Bueso-Izquierdo, Sixto Cubo-Delgado

**Affiliations:** 1 Department of Psychology and Anthropology, University of Extremadura, 06006 Badajoz, Spain; 2 Department of Educational Sciences, University of Extremadura, 06006 Badajoz, Spain

**Keywords:** Malingering, Anxiety, Depression, Fibromyalgia, Mixed anxiety–depressive disorder

## Abstract

Malingering consists of the production of false physical or psychological symptoms motivated by external incentives that are normally reproduced in pathologies that are not related to organic origin or there are no laboratory tests for their diagnosis, as is the case of mixed anxiety–depressive disorder and fibromyalgia syndrome. The objective of this research consisted of comparing the profile of simulative patients with fibromyalgia and mixed anxiety–depressive disorder to obtain a profile and facilitate its detection in initial interviews. The research was carried out with 78 patients (42 patients with fibromyalgia and 36 patients with mixed anxiety–depressive disorder) who were administered the professional's structured clinical judgment, the Beck Depression Inventory, the State-Trait Anxiety Questionnaire, and the Structured Symptom Simulation Inventory. The main obtained results show that the simulation classification proposed by the questionnaire is in the range of 66.67–80% with regard to coinciding with the judgment of experts, and people with suspicion of simulation of both groups of patients present similar characteristics. The simulators thus present incongruous responses in relation to the questionnaires, and high levels of trait anxiety, state, and depression predict the simulation of symptoms.

## Introduction

Malingering consists of the representation of false or highly exaggerated physical or psychological symptoms, motivated by external incentives to obtain financial compensation, evade criminal responsibilities, or obtain drugs [2]. Thus, there are people who can simulate or magnify their symptoms to achieve this financial or personal benefit [46]. Malingering occurs both in specific tests and in expert evaluation, and is especially aggravated in litigation or forensic evaluation situations [30, 44].

Pathologies that present a higher percentage of simulation are those in which symptoms are not directly related to the organic origin, or when there is no laboratory test or biomarker to diagnose them [23], as it happens in the case of fibromyalgia [26, 48] and mixed anxiety–depressive disorder [22, 45].

Fibromyalgia syndrome is suffered mostly by women [14, 38], with patients predominantly with low socioeconomic status and belonging to rural areas [11], although these characteristics could probably be explained by criteria used for its diagnosis [36, 52]. This syndrome has an idiopathic origin, whose main hypothesis is linked to central sensitization syndrome (SSC) [18]. Symptoms must last more than three consecutive months and usually begin erratically [53], for which only asymptomatic treatment is provided [3]. The main symptoms are chronic pain, chronic fatigue, and sleep problems [5], and a wide variety of cognitive and emotional symptoms [4, 24], such as depression, which has a prevalence of 60% in patients with this pathology [6], and anxiety, whose specific profile was described by several authors [1, 43], and its prevalence is in the range of 27–60% of patients with fibromyalgia [32].

Mixed anxiety–depressive disorder is characterized both by the presence of symptoms of anxiety (feeling nervous, anxious, or limited, unable to control worrying thoughts, fear of something terrible happening, trouble relaxing, etc.) and depression (state depressed mood or markedly decreased interest in or pleasure from activities, etc.), although no set of symptoms, taken separately, is severe, numerous, or persistent enough to warrant an independent diagnosis of depression or anxiety [51], which are also linked to central sensitization syndrome [18]. This symptomatology has high prevalence, with these being the two main mental illnesses that have the greatest socioeconomic impact [41].

A study showed that anxiety, depression, and fibromyalgia syndrome were among the main simulated pathologies according to the perception of professionals [47]. Other studies showed that the percentage of simulated or malicious symptoms in fibromyalgia ranges between 37.7 and 50% in the medical–legal context, in anxiety and depression, it is over 50% [12, 40]. The expression of any symptom is mediated by a large number of medical and social variables [19] that both directly affect the physical or cognitive aspect, and present a qualitative decrease in quality of life, and occupational and social health [39], which can sometimes increase the magnitude of certain symptoms or simulated symptoms to achieve some purpose [9, 26].

There are very few studies that analyze the responses of sham and nonsham patients in mixed anxiety–depressive disorder [10] or fibromyalgia [47], and there are no studies that analyze the common elements of simulator patients in whose pathologies there is a high prevalence of anxiety and depressive symptoms [51, 53]. Due to the above, the objective of this research is to compare the profile of patients with fibromyalgia and with mixed anxiety–depressive disorder with or without suspicions of simulation, in relation to two of the most easily simulated variables, anxiety and depression, to obtain a common profile of the patient with suspected simulation and facilitate its detection in initial interviews. The selection of the two groups for this study is due to the fact that both disorders present a high rate of anxiety and depression, without these symptoms being part of the primary pathology [49, 51].

## Materials and methods

### Participants

For the first experimental group, 42 participants diagnosed with fibromyalgia were selected, referred thanks to the Pain Unit of the Reference Hospital of the Autonomous Community of Extremadura, of which 38 were women and 4 men, with a mean age between 31 and 70 years old (M = 52.7; SD = 8.72). For the second experimental group, 36 patients diagnosed with mixed anxiety–depressive disorder referred from various centers and institutes of psychology of the Community of Extremadura were selected, of which 21 were women and 15 men, with a mean age between 28 and 67 years old (M = 49.7; SD = 10.94). The sample was marked by the following inclusion criteria: (i) being of legal age; (ii) having been diagnosed with fibromyalgia syndrome under the criteria of the American College of Rheumatology [31], and having been diagnosed with mixed anxiety–depressive disorder according to the ICD-11 criteria [51], and (iii) not have been diagnosed or have suffered from other serious health problems, such as cancer, neurological problems, neurodevelopment disorders, coronary problems, addictions, or serious accidents.

### Instruments

Semi-structured interview: an interview was conducted to collect the sociodemographic data of the patients, and the diagnostic criteria of the professional's structured clinical judgment according to the simulator/non-simulator classification of Slick et al. (1999), where two or more must present the following criteria: (i) the existence of an eternal incentive known by the evaluated subject themselves; (ii) the role of the person to evaluate as plaintiff or litigant; (iii) discordance between the magnitude and severity of the presented disease, and the presumed degree of discomfort or dysfunction; (iv) discrepancy between type of disease and described symptoms; and (v) presumed symptoms, affectation, or dysfunction in the absence of known psychopathological history.

Beck Depression Inventory (BDI), [17]: evaluates the state of mind with which the presence and severity of depression can be detected. The inventory has 21 items and was exhaustively studied, showing a Cronbach's alpha of 0.83 in the Spanish population.

State-Trait Anxiety Questionnaire (STAI) [29]: evaluates two dimensions of anxiety through 40 items. Trait anxiety refers to a personality factor that predisposes or not to suffer anxiety, and state anxiety defines the environmental factors that protect from or generate anxiety. For the Spanish version, the trait and state anxiety items obtained a Cronbach’s alpha of 0.90 and 0.94, respectively.

Symptom Simulation Structured Inventory (SIMS) [26]: measures the detection of symptoms of a psychological or neuropsychological nature, simulated or malicious, whose field of work is medicolegal, forensic, and neuropsychological evaluations. This inventory consists of 75 items and provides information on five specific scales: (i) psychosis, which assesses the degree to which the subject presents unusual or extravagant psychotic symptoms that are not typical of a real psychotic pathology; (ii) neurological impairment, which evaluates the degree of the presence of illogical or very neurologically atypical symptoms; (iii) amnesic disorders, which assesses the degree to which the patient has inconsistent memory problems with deterioration patterns caused by dysfunction or actual brain damage; (iv) low intelligence, which assesses the degree to which the subject exaggerates their intellectual deficit by wrongly answering general knowledge questions; and (v) affective disorders (AF), which assesses the degree to which the subject reports atypical symptoms of depression and anxiety. In addition, it has a global scale of suspicion of simulation with a cut-off point equal to or greater than 17. The cut-off point in the study by González Ordi and Santamaría [26] was used (simulation > 16; psychosis > 2; neurological impairment > 3; amnestic disorders > 3; low intelligence > 3; affective disorders > 7). The Spanish version was adapted with a total of 1005 subjects, where a total Cronbach’s alpha of 0.94 was obtained; for its five subscales, it was as follows: 0.85 for neurological deterioration, 0.90 for psychosis, 0.69 for low intelligence, 0.90 for amnestic disorders, and 0.65 for affective disorders.

### Procedure

Through the Pain Unit of the Autonomous Community and the different centers and institutes of psychology, patients who had voluntarily signed the informed consent were contacted and were psychologically evaluated by expert psychologists in an ideal location within the facilities of the centers. Participants were evaluated over a period of two hours, including the semi-structured interview, where the characteristics of the study, their rights as participants, and their right to leave the study with total freedom at the time they believed appropriate were clearly explained. The protocol was approved by the Doctoral Program Committee of Extremadura University with the identification code R014. All data were treated anonymously, and the research was carried out according to the ethical principles of the Declaration of Helsinki.

### Data analysis

Data were analyzed using IBM SPSS.25 to provide answers to the posed problems. To perform inferential analysis, we used the following process. The Shapiro–Wilk test was applied to contrast the null hypothesis of the normality of distribution, and the Rachas test to test the null hypothesis that the theoretical distribution in the population was random. Levene's test was carried out to contrast the null hypothesis related to the equality of variances between the different analyzed variables. After these analyses, the contingency coefficient statistical model was applied to observe the relationship between the suspicion of simulation, age, and economic level, and the prior clinical judgment of the professional in the two groups of patients, The Mann–Whitney *U* statistical model was used to observe the relationship of the scores between the subjects who presented or not a simulation of symptoms in both populations. The nonparametric statistical model of Spearman's rho was used to analyze the relationship between suspicion of simulation with its dimensions, and the variables of anxiety and depression for both groups of patients. Lastly, multiple regression was performed to detect possible predictors of simulated symptoms.

## Results

After the semi-structured interview, sample characteristics were collected and are shown in Table 1.

**Table 1:** Characteristics of the patients

Characteristics	Fibromyalgia (*N* = 42)	Mixed anxiety-depressive disorder (*N* = 36)
	*N* (%)	*N* (%)
Age range		
30/50	15 (35.71)	18 (50.00)
50/65	23 (54.76)	14 (38.88)
> 65	4 (9.53)	4 (11.11)
Education level		
7–12 primary	12 (28.57)	10 (27.77)
13–16 high school	7 (16.67)	12 (33.33)
17–18 bachelorship	15 (35.71)	9 (25)
19–21 university	8 (19.04)	5 (13.89)
Sick leave		
Never	20 (47.62)	20 (55.56)
Yes, at this moment	8 (19.04)	9 (25.00)
Yes, previously	14 (33.34)	7 (19.44)
Civil status		
Married	29 (69.05)	21 (58.33)
Divorcee/divorced	8 (19.04)	6 (16.67)
Widow/er	2 (4.76)	2 (5.56)
Single	3 (7.15)	7 (19.44)
Economic level^a^		
< 6	15 (35.71)	8 (22.22)
6–12	11 (26.19)	7 (19.44)
12–24	11 (26.19)	11 (30.56)
> 24	5 (11.91)	10 (27.78)
Suspicion of malingering		
Yes	11 (26.19)	12 (33.33)
No	31 (73.81)	24 (66.66)
Expert clinical judgment^b^		
Yes	10 (23.81)	15 (41.67)
No	32 (76.19)	21 (58.33)

^a^Economic level: × 1000

^b^The diagnostic criteria of the professional's structured clinical judgment (Slick et al. 1999)

According to the descriptive analysis in relation to variables suspected of simulating symptoms and age, only 11 of the 42 subjects with fibromyalgia and 12 of the 36 with mixed anxiety–depressive disorder exceeded 16 points on the global scale that indicates signs or suspicions of simulation symptoms. After analysis, no significant relationship could be found between the suspicion of simulation, age, economic level, and medical loss for both populations, but between the suspicion of simulation of the SIMS and the clinical judgment of the professional for patients with fibromyalgia (< 0.001) and mixed anxiety–depressive disorder (< 0.001), in which the coincidence of both positive evaluations ranged between 80 and 66.67% (Table 2).

**Table 2:** Cross table of the variables age and suspected simulation of symptoms

Fibromyalgia					
Suspicion of simulation	No (%) 31 (73.8)	Yes (%) 11 (26.2)	Total (%) 42 (100)	*c*	*p*
Age range					
30/50	10 (66.67)	5 (33.33)	15 (100)	0.057	0.121
50/65	17 (73.9)	6 (26.1)	23 (100)		
> 65	4 (100)	0 (0)	4 (100)		
Nivel económico^a^					
− 6	12 (80)	3 (20)	15 (100)	0.263	0.067
6–12	7 (63.6)	4 (36.4)	11 (100)		
12–24	8 (72.72)	3 (27.27)	11 (100)		
> 24	4 (80)	1 (20)	5 (100)		
Sick leave					
Never	16 (80.00)	4(20.00)	20 (100)	0.279	0.194
Yes, at this moment	7 (87.5)	1(12.5)	8 (100)		
Yes, previously	8 (57.14)	6(42.86)	14 (100)		
Expert clinical judgment^b^					
Yes	2 (20)	8 (80)	10 (100)	0.655	< 0.001
No	29 (90.63)	3 (9.37)	32 (100)		

Mixed anxiety-depressive disorder					
Suspicion of simulation	No (%) 24 (66.66)	Yes (%) 12 (33.33)	Total (%)36 (100)	c*	*p*
Age range					
30/50	13 (72.22)	5 (27.78)	18 (100%)	0.262	0.350
50/65	9 (64.28)	5 (35.74)	14 (100%)		
> 65	2 (50.00)	2 (50.00)	4 (100%)		
Nivel económico^a^					
-6	6 (80.00)	2 (20.00)	8 (100%)	0.192	0.248
6–12	4 (57.14)	3 (42.86)	7 (100%)		
12–24	8 (72.73)	3 (27.27)	11 (100%)		
> 24	6 (60.00)	4 (40.00)	10 (100%)		
Sick leave					
Never	17 (85.00)	3 (15.00)	20 (100%)	0.258	0.283
Yes, at this moment	4 (44.44)	5 (55.56)	9 (100%)		
Yes, previously	3 (42.86)	4 (57.14)	7 (100%)		
Expert clinical judgment^b^					
Yes	5 (33.33)	10 (66.67)	15 (100%)	0.751	< 0.001
No	19 (90.48)	2 (9.52)	21 (100%)		

The correlation is significant at the 0.05 level (bilateral)

*c* contingency coefficient

^a^Economic level: × 1000

^b^The diagnostic criteria of the professional's structured clinical judgment (Slick et al. 1999)

**Fig. 1:**
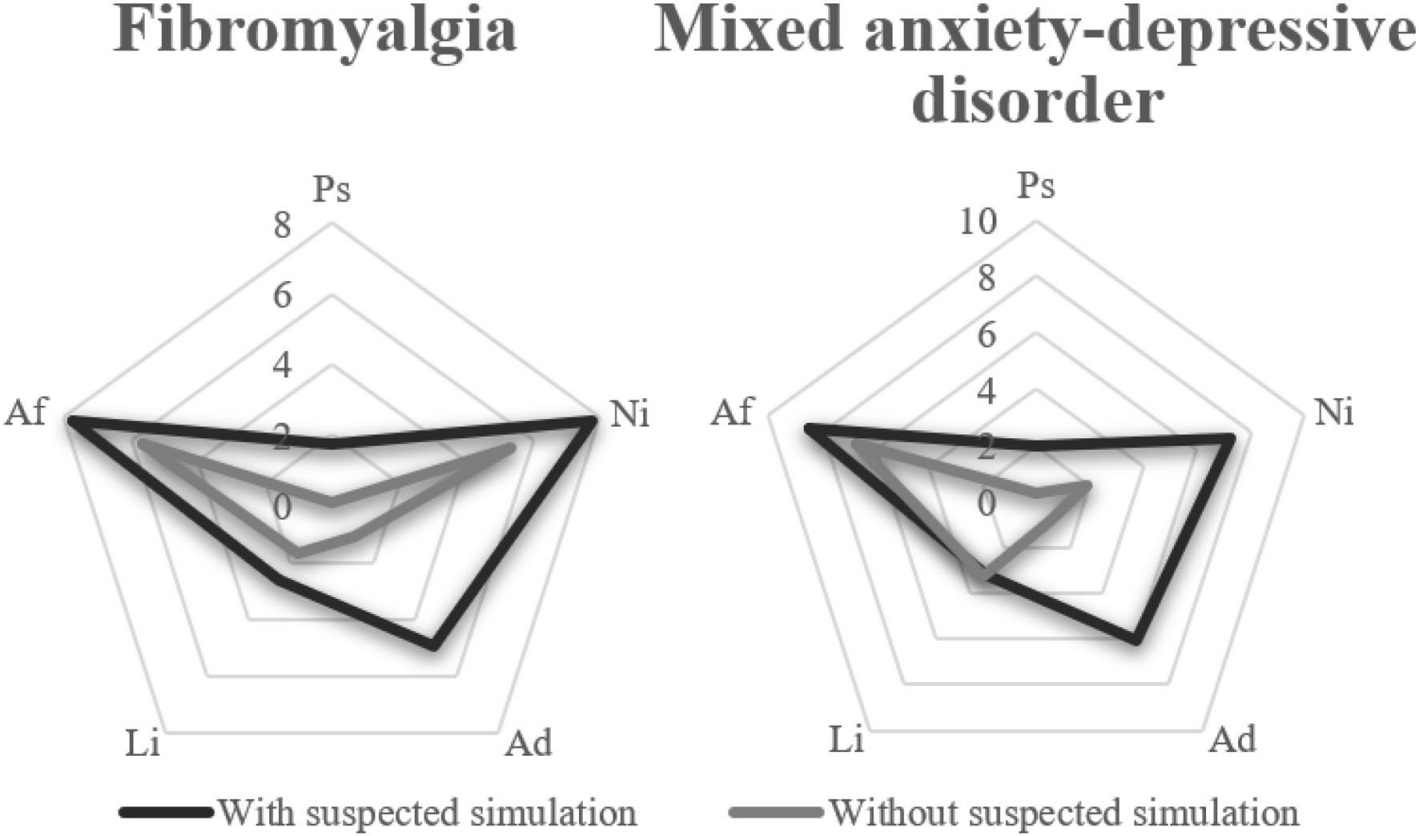
Comparison of the results of the SIMS dimensions scores in a patient with and without suspected simulation diagnosed with fibromyalgia and mixed anxiety-depressive disorder*.* *Ps* psychosis, *Ni* neurological impairment, *Ad* amnesic disorders, *Li* low intelligence, *Af* affective disorders

If we observe the means of all the subjects with fibromyalgia who presented suspicion of simulation, the highest average corresponds to neurological deterioration, followed by affective disorders, amnestic disorders, low intelligence, and psychosis, remaining only below the psychosis cut-off point and low intelligence. This pattern was very similarly repeated in the scores of the group that did not present suspicion of simulation, but with lower scores, where only the cut-off point for the neurological impairment subscales was exceeded. In the case of patients with mixed anxiety–depressive disorder, all scores except for low intelligence were higher in the group with suspected simulation. In this group, only the psychosis subscale was below the cut-off point, while in the group without suspicion of simulation, the cut-off point was not exceeded in the psychosis, neurological deterioration, and amnestic disorders subscales (Fig. 1).

The comparison of means shows that, after applying the nonparametric Mann–Whitney U test, significant differences existed when comparing the two groups, both for the total symptom simulation variable (*p* < 0.001) and for the dimensions of psychosis (*p* = 0.004), neurological deterioration (*p* = 0.001), amnestic disorder (*p* = 0.001), and affective disorder (*p* = 0.017) in patients with fibromyalgia, with low intelligence being the only one that did not show significant differences (*p* = 0.163). In patients with mixed anxiety–depressive disorder, significant results were shown when comparing the two groups for the total symptom simulation variable (*p* < 0.001), and for psychosis (*p* = 0.028), neurological deterioration (*p* < 0.001), and disorder dimensions of amnesic (*p* < 0.001), with low intelligence (*p* = 0.878) and affective disorder (*p* = 0.174) being the only ones that did not show significant differences.

However, the entire results between patients with fibromyalgia and mixed anxiety–depressive disorder verified that there were no significant differences on the global scale, and in none of the subscales except neurological deterioration in the group of patients with suspicion of simulation (*p* < 0.001); in patients without simulation, there were no significant differences (*p* = 0.474) in this subscale.

In relation to the depression and anxiety variables, comparing the two groups of patients with fibromyalgia shows that they did not show significant differences in the BDI (*p* = 0.927) between them; for the STAI trait (*p* = 0.029) and STAI state (*p* < 0.001), there were significant differences between people with and without suspected simulation. In patients with mixed anxiety–depressive disorder, significant differences were shown among BDI (*p* < 0.001), STAI trait (*p* < 0.001), and STAI state (*p* = 0.045) in the same way that significant differences were shown when comparing the patients of both groups (fibromyalgia and mixed anxiety–depressive disorders) and their scores on the BDI (*p* < 0.001), the STAI trait (*p* = 0.014) and STAI state (*p* = 0.002) of the group without suspicion of simulation, and in the BDI (*p* < 0.001), the STAI trait (*p* < 0.001) and STAI status (*p* = 0.023) of the group with suspected simulation (Table 3).

**Table 3:** Means and number of cases of the symptom simulation questionnaire and its dimensions in the SIMS questionnaires

	Means (*σ*) of the total scores by group								Differences between patients with fibromyalgia and mixed anxiety-depressive disorder			
	Fibromyalgia				Mixed anxiety-depressive disorder				Without suspected simulation		With Suspected simulation	
	Without suspected Simulation (*n* = 31)	With Suspected simulation (*n* = 11)	*U*^a^	*p*^b^	Without suspected simulation (*n* = 24)	With Suspected simulation (*n* = 12)	*U*^a^	*p*^b^	*U*^a^	*p*^b^	*U*^a^	*p*^b^
Simulation	13.94 (2.63)	24.79 (6.84)	20.4000	< 0.001	12.32 (2.98)	25.34 (9.75)	32.00	< 0.001	92.00	0.542	72.00	0.554
Psychosis	0.08 (0.28)	1.76 (1.93)	69.000	0.004	0.35 (0.452)	1.98 (1.57)	35.00	0.028	62.00	0.274	82.00	0.364
Neurological impairment	5.32 (1.54)	7.79 (2.54)	51.000	< 0.001	1.87 (1.97)	7.24 (2.48)	26.50	< 0.001	36.00	< 0.001	69.00	0.474
Amnesic disorders	1.11 (1.33)	4.93 (2.30)	40.500	< 0.001	0.83 (1.39)	6.04 (2.87)	28.50	< 0.001	87.00	0.472	65.00	0.175
Low intelligence	1.67 (1.70)	2.59 (1.70)	100.00	0.163	3.24 (1.67)	3.13 (1.88)	147.00	0.878	82.00	0.190	76.00	0.491
Affective disorders	5.65 (1.56)	7.72 (1.67)	82.000	0.017	6.69 (1.76)	8.48 (1.94)	92.00	0.174	81.00	0.182	46.00	0.113
BDI	23.38 (9.06)	25.18 (15.13)	156.500	0.927	31.49 (8.03)	38.24 (13.34)	38.00	< 0.001	37.00	< 0.001	52.00	< 0.001
STAI—rasgo	29.91 (12.42)	35.28 (9.06)	87.500	0.029	36.91 (12.42)	42.74 (14.52)	42.00	< 0.001	47.00	0.014	34.00	< 0.001
STAI—estado	27.36 (8.76)	35.93 (9.89)	37.500	< 0.001	34.07 (14.76)	39.46 (11.88)	65.50	0.045	72.50	0.002	47.00	0.023

Cut-off points (Simulation > 16; Psychosis > 2; Neurological impairment > 3; Amnestic disorders > 3; Low intelligence > 3; Affective disorders > 7) used in González Ordi and Santamaría [26]

^a^*U*: Mann–Whitney *U*

^b^The correlation is significant at the 0.05 level

Analysis of correlations between the simulation of symptoms of anxiety and depression showed that, for patients with fibromyalgia in the group without suspicions of simulation, only the dimensions of amnestic disorders with state anxiety (*p* = 0.003) and with trait anxiety were significant (*p* = 0.005). In the group with suspected simulation, the relationships between the simulation variable with depression (p < 0.001) and state anxiety (*p* = 0.026), and trait anxiety (*p* = 0.030) were significant. Furthermore, the psychoticism dimension was significant with depression (*p* = 0.042) and state anxiety (*p* = 0.026). The amnestic disorder dimension also showed significance with depression (*p* = 0.002) and state anxiety (*p* = 0.002). In this group, correlation between depression, and state (*p* < 0.001) and trait (*p* < 0.001) anxiety was significant.

For patients with mixed anxiety–depressive disorder without suspicion of simulation, only the relationship between the simulation variable with trait anxiety (*p* < 0.001), and amnestic disorders with trait anxiety (*p* = 0.026) and with state anxiety (*p* < 0.001) were significant). For patients with suspected simulation, there was a positive relationship between simulation with depression (*p* < 0.001), simulation with state anxiety (*p* < 0.001), and with trait anxiety (*p* < 0.001), and amnestic disorders with state anxiety (*p* < 0.001). In relation to the variables of depression with state anxiety and depression with trait anxiety, they were significant (*p* < 0.001) for both groups (with and without suspected simulation) (Table 4).

**Table 4:** Relationship between suspected simulation, anxiety and depression

Variables	Fibromyalgia					Mixed anxiety-depressive disorder			
	Without suspected simulation (*N* = 31)		With suspected simulation (*N* = 11)		Without suspected simulation (*N* = 24)			With suspected simulation (*N* = 12)	
	Rho	(*p*)	Rho	(*p*)	Rho		(*p*)	Rho	(*p*)
Simulation—depresión	0.257	0.389	0.612	< 0.001	− 0.084		0.857	0.572	< 0.001
Ps—depression	0.212	0.441	0.380	0.042	0.274		0.574	0.147	0.678
Ni—depression	0.374	0.263	0.339	0.072	0.286		0.247	0.247	0.302
Ad—depression	0.458	0.298	0.550	0.002	0.258		0.385	0.427	0.589
Li—depression	0.869	0.071	0.083	0.668	− 0.089		0.751	0.274	0.241
Af—depression	− 0.162	0.652	0.280	0.144	0.236		0.339	0.196	0.789
Simulation—state anxiety	0.594	0.341	0.382	0.041	0.247		0.478	0.098	< 0.001
Ps—state anxiety	0.287	0.402	0.413	0.026	0.236		0.578	0.347	0.214
Ni—state anxiety	0.352	0.358	0.123	0.525	0.427		0.259	0.874	0.671
Ad—state anxiety	0.747	0.003	0.560	0.002	0.649		< 0.001	0.657	< 0.001
Li—state anxiety	− 0.043	0.569	0.113	0.561	− 0.147		0.458	0.347	0.347
Af—state anxiety	0.257	0.578	0.019	0.921	0.078		0.985	0.478	0.347
Simulation—trait anxiety	0.675	0.744	0.173	0.030	0.688		< 0.001	0.120	< 0.001
Ps—trait anxiety	0.359	0.298	0.273	0.152	0.178		0.780	0.324	0.378
Ni—trait anxiety	0.365	0.226	0.023	0.904	0.147		0.589	0.357	0.689
Ad—trait anxiety	0.735	0.005	0.200	0.297	0.478		0.026	0.097	0.895
Li—trait anxiety	0.656	0.654	0.194	0.314	0.458		0.274	0.214	0.387
Af—trait anxiety	− 0.125	0.936	0.921	0.578	0.257		0.375	0.125	0.287
Depression—trait anxiety	0.562	0.936	0.481	< 0.001	0.784		< 0.001	0.614	< 0.001
Depression—trait anxiety	0.137	0.936	0.542	< 0.001	0.689		< 0.001	0.578	< 0.001

The correlation is significant at the 0.05 level (bilateral)

*Rho* Spearman's rho, *Simulation* suspected simulation of symptoms, *Ps* Psychosis, *Ni* neurological impairment, *Ad* amnesic disorders, *Li* low intelligence, *Af* affective disorders

Regarding multiple regression analysis, significant results were only obtained for the groups of patients with suspected simulation, both for fibromyalgia and for mixed anxiety–depressive disorders. Symptom simulation in fibromyalgia patients depends 50.1% on trait anxiety (*p* < 0.001), 57.8% on state anxiety (*p* < 0.001), and 51.8% on depression (*p* = 0.042) in patients with fibromyalgia who had exceeded the cut-off to present suspicion of simulation. Similarly, the simulation of symptoms in patients with mixed anxiety–depressive disorder depends 77.4% on trait anxiety (*p* < 0.001), 86.9% on state anxiety (*p* < 0.001), and 63.4% on depression (*p* = 0.024) in patients with fibromyalgia who had exceeded the cut-off to present suspicion of simulation (Table 5).

**Table 5:** Predictors of malingering

	Coefficients^a^	*R*^2^	Beta	*t*	*p*
Fibromyalgia					
With suspected simulation (*N* = 11)	Trait anxiety^b^	0.501	0.647	3.445	< 0.001
	State anxiety^b^	0.578	0.686	4.957	< 0.001
	Depression^b^	0.518	0.358	1.872	0.042
Without suspected simulation (*N* = 29)	Trait anxiety^b^	0.548	0.102	0.184	0.859
	State anxiety^b^	0.474	0.479	0.904	0.396
	Depression^b^	0.004	− 0.012	− 0.035	0.973
Mixed anxiety–depressive disorder					
With suspected simulation (*N* = 12)	Trait anxiety^b^	0.774	0.587	4.125	< 0.001
	State anxiety^b^	0.869	0.659	4.158	< 0.001
	Depression^b^	0.634	0.672	4.378	0.024
Without suspected simulation (*N* = 24)	Trait anxiety^b^	0.327	0.324	1.745	0.279
	State anxiety^b^	0.294	0.214	1.256	0.128
	Depression^b^	0.389	0.245	1.483	0.263

^a^Dependent variable: symptom simulation

^b^Predictors: (constant), trait anxiety, state anxiety, depression

## Discussion

In total, 27.5% of the patients with fibromyalgia had a suspicion of simulation, which is consistent with the studies by Capilla-Ramírez et al. [12] and Mittenberg et al. [40], where 22.5% and 37.8% of simulation, respectively, were shown. In patients with mixed anxiety–depressive disorder, the percentage was 33.33%, which exceeded that stated by Mittenberg et al. [40], who reported that 16.08% of depressive syndromes diagnosed in litigation were simulated, but it is below 53.85% of patients with mixed anxiety–depressive disorder, simulating Blasco-Saiz and Pallardó-Durá [10]. The relationship between the suspicion of simulation, age, economic level, and the situation of medical leave cannot affirm that there is a significant relationship between these variables after the application of the test in our population, despite the fact that there are studies that associate age at the diagnosis of fibromyalgia syndrome [52] and precarious economic conditions to simulation [27, 47]. In connection with the simulation criteria, the SIMS instrument ratified the proportional professional clinical judgment in 66.67–80% of the cases. These results coincide with those presented by López-Miquel and Pujol-Robinat [34], in whose research it was ratified in 69% of the cases, which shows that SIMS is reliable to detect simulation.

In our study, significant results were shown in the global dimension of SIMS in fibromyalgia, which contradicts what was stated by Capilla-Ramírez et al. [13], because they did not find significant discriminant scores between patients with and without suspected simulation. In addition, all dimensions of the questionnaire showed higher significant scores in the simulation group with the exception of low intelligence, which is consistent because fibromyalgia does not present symptoms of an intellectual nature. However, the psychosis dimension is also not a symptom that is present in its diagnosis, and shows higher significant scores in simulation, which would indicate that simulators refer to extravagant and atypical symptoms in this disease to obtain recognition, as indicated by Bass and Wade [8]. Consequently, fibromyalgia patients with suspected simulation score being significantly more in the affective disorders and neurological deterioration dimensions is within expectations, since both dimensions are included in the spectrum of fibromyalgia syndrome [50]. Although these dimensions evaluate atypical symptoms, the peculiarities of each disorder should be taken into account because not all exaggerations or magnifications of symptoms are a simulation [28].

In fact, in the neurological impairment dimension, which refers to somatic-type simulations [13], there were significant differences with higher scores in fibromyalgia than those in mixed anxiety–depressive disorder in patients without suspected simulation. This could be because fibromyalgia syndrome is traditionally associated with somatic symptoms [7, 33] and they present very atypical neurological symptoms due to their nature or form of appearance, as already indicated Wolfe et al. [53]. These differences did not occur in the group with suspected simulation, given that both disorders similarly scored to that reported by Blasco-Saiz and Pallardó-Durá [10]. In their study, there seems to have been a pattern of responses of somatic symptoms that was repeated in simulators. According to López-Miquel and Pujol-Robinat [34], this scale is one with the greatest discriminatory power between simulator and non-simulator conditions.

In the mixed anxiety–depressive disorder group, significant differences were also shown in the global simulation scale and in all subscales except low intelligence, with low scores in both groups, and affective disorders with high scores in both because this disorder presents affective symptoms in its diagnosis [16]. The same profile was found as in the study by Blasco-Saiz and Pallardó-Durá [10], which indicated that there is a consistent pattern of exaggeration with this type of affective disorders by simulators.

Other studies that corroborate our results were those carried out by Monaro et al. [41], who demonstrated that simulating individuals reported a greater number of depressive and nondepressive symptoms than those participants without simulation, and the study by Martínez et al. [37] who stated that the higher the anxiety levels in patients with fibromyalgia were, the higher the score they presented in the depression variable. In our study, these results were also extrapolated to patients with mixed anxiety–depressive disorder, which shows great similarity in their responses to affective symptoms.

We can thus conclude that high levels of trait anxiety, state, and depression predict the simulation of symptoms only in the group of patients with suspected simulation with fibromyalgia and mixed anxiety–depressive disorder. This is another sign that anxiety and depressive symptoms are easily simulated and highly recurrent for the expression of malicious symptoms or the magnification of symptoms [15, 25, 47].

Low and Schweinhardt [35] described that factors that predispose to developing depression from a very early age are also risk factors for suffering from fibromyalgia. All similarities that we found between simulator profiles in both disorders, as described in other investigations [10, 26], could be explained by the amount of affective symptoms. Difficult-to-verify common problems presented by both pathologies were included within the SSC [4, 20, 21, 54, 55]. Both disorders share common elements, which is why the simulating subjects of both pathologies present similar characteristics in terms of their simulation responses to symptoms.

Limitations of the study include the difficulty of finding patients who met the inclusion criteria, the sample size, and the obtained responses to the questionnaires could be mediated by the appearance of distortions or response biases, such as central tendency and social desirability. Furthermore, the sample was not selected through a judicial or forensic route, but clinically, so it would be interesting for future studies to include a specific sample selected within the forensic field, and to use other simulation evaluation methods complementary to SIMS and the evaluation of experts [27, 42].

## Conclusion

The findings of this work may contribute to outlining the simulatory profile of patients with fibromyalgia and mixed anxiety–depressive disorder, thus improving the future treatment of these patients and those with legitimate symptoms.

- People with suspected simulation diagnosed with fibromyalgia and with mixed anxiety–depressive disorder present similar characteristics in their simulation responses.
- Fibromyalgia patients who do not present suspicion of simulation obtain high scores in anxiety and neurological deterioration, while those who do present suspicion also score high in amnestic disorders.
- Simulative mixed anxiety–depressive patients show illogical neurological symptoms, which were associated with somatic symptoms.
- High levels of trait, state, and depression anxiety predict symptom simulation.

## Data Availability

All data generated or analyzed during this study are included in this published article.
